# New Appliances and Things Medical

**Published:** 1901-08-31

**Authors:** 


### NEW APPLIANCES AND THINGS MEDICAL.

[We shall be glad to receive, at our Office, 28 & 29 Southampton Street, Strand, London, W.C., from the manufacturers, specimens of all new preparations and appliances which may be brought out from time to time.]

### CONDENSED MILK.

(Milkmaid Brand.)

(Anglo-Swiss Condensed Milk Company, 10 Mark Lane, London, E.C.)

We have received two samples of the condensed milk prepared by the above firm—one of English and the other of Swiss manufacture. Both of them are excellent examples of what a condensed milk should be; that is to say as compared with the albuminous contents they both contain the same relative proportion of cream as does good average fresh milk. By diluting with a sufficiency of water the resultant mixture closely approximates to boiled cows' milk to which has been added a certain proportion of sugar. Owing to the convenient manner in which this condensed milk can be applied to the purpose of infant feeding it has become one of the most popular foods for this purpose, not only among the poorer classes, but also among the well-to-do in those cases in which fresh cows' milk has been found to disagree. We would strongly recommend those who adopt this method of feeding to follow very closely the direction supplied with the milk, and, further, in order that the tendency to scurvy which is manifested in infants from the continued use of a preserved food may be effectually controlled, we recommend that occasionally a little fresh cream, fresh meat-juice, or the juice of an orange or a few grapes should be given independently of the daily supply of condensed milk.

### IMPROVED POCKET SPITTOON.

(W. H. Bailey and Son, 38 Oxford Street, London, W.)

This new pocket spittoon is an improvement of the original flask invented by Dittweiler. It is oval in shape, and well adapted to slip easily into the pocket, it is made of blue glass, and fitted with stout metal screw caps, one at each end, with indiarubber washers. One of the chief advantages is that the larger screw cap, which closes the orifice through which the sputum enters the receptacle, can be taken off and reapplied with a minimum of trouble. The price is only 1s., a great advantage in the case of hospitals and institutions where large numbers have to be supplied. In every way this new spittoon is superior to any one with which we are acquainted.

